# Stunting among Preschool Children in India: Temporal Analysis of Age-Specific Wealth Inequalities

**DOI:** 10.3390/ijerph17134702

**Published:** 2020-06-30

**Authors:** Sunil Rajpal, Rockli Kim, William Joe, S.V. Subramanian

**Affiliations:** 1 Institute of Health Management Research, IIHMR University, Jaipur 302029, India; sunil@iihmr.edu.in; 2 Division of Health Policy and Management, College of Health Sciences, Korea University, Seoul 02841, Korea; 3 Department of Public Health Sciences, Graduate School, Korea University, Seoul 02841, Korea; 4 Harvard Center for Population and Development Studies, Cambridge, MA 02138, USA; 5 Population Research Centre, Institute of Economic Growth, Delhi 110007, India; william@iegindia.org; 6 Department of Social and Behavioral Sciences, Harvard T.H. Chan School of Public Health, Boston, MA 02115, USA

**Keywords:** stunting, undernutrition, age, critical periods, nutrition interventions, socioeconomic status

## Abstract

Adequate nutritional intake for mothers during pregnancy and for children in the first two years of life is known to be crucial for a child’s lifelong physical and neurodevelopment. In this regard, the global nutrition community has focused on strategies for improving nutritional intake during the first 1000 day period. This is largely justified by the observed steep decline in children’s height-for-age z scores from birth to 23 months and presumed growth faltering at later ages as a reflection of earlier deprivation that is accumulated and irreversible. Empirical evidence on the age-stratified burden of child undernutrition is needed to re-evaluate the appropriate age for nutrition interventions to target among children. Using data from two successive rounds of National Family Health Surveys conducted in 2006 and 2016, the objective of this paper was to analyze intertemporal changes in the age-stratified burden of child stunting across socioeconomic groups in India. We found that child stunting in India was significantly concentrated among children entering preschool age (24 or above months). Further, the temporal reduction in stunting was relatively higher among children aged 36–47 months compared to younger groups (below 12 and 12–23 months). Greater socioeconomic inequalities persisted in stunting among children from 24 months or above age-groups, and these inequalities have increased over time. Children of preschool age (24 or above months) from economically vulnerable households experienced larger reductions in the prevalence of stunting between 2006 and 2016, suggesting that policy research and strategies beyond the first 1000 days could be critical for accelerating the pace of improvement of child nutrition in India.

## 1. Introduction 

It is widely acknowledged that health and nutrition-related interventions during the first 1000 days of life are critical to arrest the momentum of growth faltering among children [1,2,3]. Despite considerable policy efforts, India is yet to witness significant progress in reducing the incidence of child undernutrition in this age group (0–23 months). The National Family Health Surveys (NFHS) report significantly high stunting prevalence of 39% and 33% among children aged 0–23 months during 2006 and 2016, respectively [4,5]. A slow pace of improvement in nutritional status during the first 1000 days has adverse consequences in terms of both the lack of recovery and new incidences of growth faltering that are reflected in the higher prevalence of anthropometric failures in later years. For instance, in 2016, the stunting prevalence during the first 1000 days (0–23 months) and the preschool days (24–59 months) was 33% and 42%, respectively. In absolute terms, this cumulates into millions of children who are at risk of sub-optimal physical and cognitive development.

The programmatic focus on the first 1000 days—for instance in the case of the POSHAN (Prime Minister’s Overarching Scheme for Holistic Nourishment) Abhiyaan in India—is essentially based on the fact that anthropometric failures (such as low height-for-age standardized z-scores) in developing countries exhibit rapid deterioration from birth to 23 months and remain more or less static thereafter [6,7,8]. Accordingly, a large number of nutrition interventions in India and elsewhere are devised to focus on children 0–23 months, thereby undermining the potential for nutritional improvements of children 24–59 months. This presumption implies that anthropometric failures are not only irreversible but also new incidences of undernutrition at later ages result solely from earlier deprivation. However, it is apparent that children with normal growth in the first two years of life are at risk of growth faltering in later years [9]. At the same time, while some children may suffer from irreversible forms of undernutrition, others can display substantial catch-up growth potentials [10,11,12]. This evidence indicate that, in order to ensure long-term development, the ongoing policy efforts focused on the first 1000 days should reasonably expand focus toward preschool children (24–59 months) as well [13,14,15].

Explicit attention on patterns and improvements in age-specific anthropometric failure by socioeconomic status assumes salience for two reasons. First, poor households may endure a disproportionately higher burden of nutritional failures among the preschool children because of high fertility rates and higher shares in the child population. Second, the pace of nutritional improvements can vary across socioeconomic groups and may differentially elevate the relative risk of anthropometric failure by age groups among the poor. In fact, household wealth is among the strongest correlate of child undernutrition; hence, a direct focus on this indicator is desirable to investigate the age-specific prevalence and associated reductions [16,17,18]. Yet, to our knowledge, no comprehensive evidence exists on changes over time in socioeconomic patterning for age-specific anthropometric failure.

With this motivation, this paper examines two successive rounds of National Family Health Surveys (NFHS) conducted during 2006 and 2016, respectively, to develop insights on the age-specific burden of stunting among Indian children. The analysis develops mutually exclusive intersectional groups-cross-classified by age and household wealth quintiles-to examine the dynamics of the burden of child stunting in India. Multivariate econometric analysis is used to discern mutual associations of child stunting and to describe the age-specific prevalence among children from economically vulnerable households. The results are expected to inform policy discussion around the relevance of age-specific nutritional interventions in India, especially in terms of the inequality by household wealth. 

## 2. Data Source and Methods 

### 2.1. Survey Data and Study Population 

**Figure A1:**
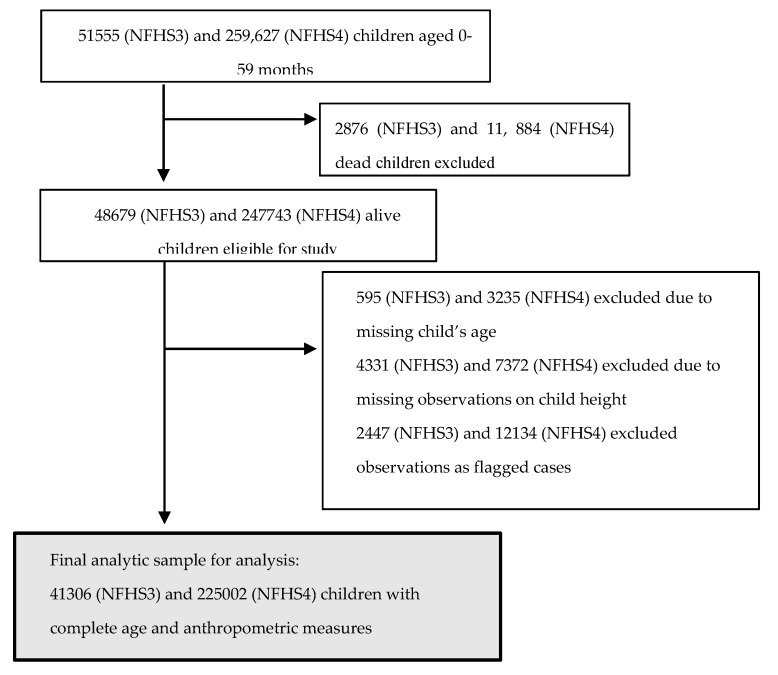
Flow diagram showing exclusions and final sample sizes of the study population, Indian National Family Health Survey 2005–2006 and 2015–2016.

The study is based on the data from two successive rounds of NFHS (2006 and 2016), which provide nationally representative information from all the States and Union Territories (UTs) of India. The sampling frame for the NFHS 2006 and 2016 was based on the Census of India, 2001, and 2011, respectively. The NFHS survey design allows for the estimation of growth faltering among children for each of the States and UTs and by rural and urban areas separately. Data in the NFHS was obtained from a two-stage stratified sampling frame. The villages (for rural areas) and Census Enumeration Blocks (for urban areas) served as primary stage units. In the second stage, households were selected for the survey from each primary sampling unit/village/block based on the probability systematic sampling. The NFHS 2006 and 2016 provide data on 51,555 and 259,627 children, respectively, aged 0–59 months. After excluding 2876 (NFHS 2006) and 11884 (NFHS 2016) dead children, those missing information on the child’s age (595 and 3235 children for NFHS 2006 and 2016, respectively) and anthropometric measures (4331 for NFHS 2006 and 7372 for NFHS 2016), and flagged observations (2447 and 12,134 from NFHS 2006 and 2016, respectively), a final analytic sample of 41306 and 225,002 children from the NFHS 2006 and NFHS 2016, respectively, was used for the analysis (Figure A1 Appendix A).

### 2.2. Primary Outcomes and Covariates

Based on the World Health Organization (WHO) child growth reference standards, a binary outcome variable for child stunting was constructed [19]. We also considered severe stunting as a secondary outcome indicator. The NFHS provides standard information on child’s height. While standing height was taken for children aged 24–59 months, the recumbent length was measured in the case of younger children (below the age of 24 months). The raw height measures were transformed into age-and sex-specific z-scores using WHO child growth standards. Stunting was defined as height-for-age z-scores less than -2 SD (-3 SD for severe stunting). The analysis primarily focuses on the prevalence and distribution of outcome variables across children’s age groups and household wealth quintiles. To elaborate, at the household level, wealth index was taken as the proxy indicator for a household’s income. The wealth quintiles provided by the survey data were based on principal component analyses of household assets and wealth characteristics [5]. The NFHS provided self-reported binary information on an exhaustive list of the possession of 32 assets, and based on that, wealth scores were obtained via principal component analysis [5].

### 2.3. Statistical Analysis

The primary analysis was stratified into five age-groups of one year each-0–11 months, 12–23 months, 24–35 months, 36–47 months, and 48–59 months. For state-level estimates, the age stratification was done as children 0-23 months and 24–59 months considering state-level sample size. First, the prevalence and distribution of child stunting across age groups and wealth quintiles were descriptively assessed through cross tables and diagrams. It is worth noting that the prevalence estimates were computed using analytical weights (aweights). Second, multilevel logistic regression analysis was conducted to understand the independent and multivariate-adjusted associations between children’s age and risk of stunting. In a multivariate framework, we included household’s wealth quintile, social group, religion, place of residence, maternal height, and maternal education. The multilevel models adjusted for the state, and village/block (primary sampling unit) level random effects. Initially, the association of stunting was assessed with a child’s age and sex only (Model 1). Further, the regression was mutually adjusted for all the socioeconomic variables, including wealth quintiles, social groups, religion, and place of residence (Model 2). Finally, the third model additionally adjusted for maternal covariates, including height, education, and body mass index (BMI) (Model 3). Lastly, we ran a separate multivariate logistic regression model with a mutually exclusive intersectional group defined by child’s age and wealth quintiles to further the understanding of the socioeconomic patterning in the age-specific burden of anthropometric failures. Therefore, wealth quintile and age groups taken together formed 25 intersectional groups (five age groups × five wealth quintiles). This model was fully adjusted for household’s socioeconomic characteristics and maternal covariates mentioned above. The regression estimates are reported in the form of odds ratios (OR) along with a 95% confidence interval. All the analyses were performed using Stata 15.0 [20].

## 3. Results

The prevalence of stunting among children (below five years) across five age-groups in India is presented in Table 1. Compared to children below 12 months, the estimates elicit a relatively higher absolute decline in the prevalence of stunting among older age groups between 2006 and 2016. For example, stunting prevalence among children aged 12 to 23 months has declined by about 10 percentage points between 2006 (52.4 percent) and 2016 (42.7 percent), whereas those below 12 months have experienced an absolute decline of 3.3 percentage points (Table 1). Even a higher absolute decline of 12.3 percentage points (from 55.9 percent in 2006 to 42.7 percent in 2016) was observed among children belonging to the 24–35 months age bracket. A similar level of reductions in stunting was observed for children older than 36 months. In relative terms, stunting declined by 13 percent among children below 12 months, 18.5 percent among 12–23 months, and 21.1 percent among those aged 48–59 months. A consistent pattern was noted for severe stunting, with relatively larger reductions observed among children entering preschool age (24 months or above) (Table A1 Appendix B). When classified into two age-groups (i.e., 0–23 and 24–59 months), the prevalence of stunting was about 10 percentage points higher among those between 24–59 months (42.0 percent) than those below the age of 24 months (32.6 percent) in 2016 (Table A2 Appendix C).

Stunted children were evenly distributed across all age-groups of above 12 months. About 22.3 percent and 23.8 percent of stunted children in India were from 24–35 months and 36–47 months of age cohort in 2016. It may also be noted that this pattern was observed to be about the same between 2006 and 2016. Besides, no significant change in the concentration of stunting across all age groups was observed between 2006 and 2016. The distribution pattern was similar for severe stunting as well. 

The prevalence of stunting was substantially higher among preschool children (24–59 months) from the poorest households (Table 2). In 2016, more than half of children aged 36–47 months (59.0 percent) and 48–59 months (54.5 percent) from the lowest wealth quintile were stunted. Even among the richest households, stunting prevalence was higher among children of preschool age (24.4 percent among 36–47 months and 20.3 percent among 48–59 months) compared to those below 12 months (14.8 percent). However, among children between 12–23 months from the richest households, the prevalence of stunting was 26.6 percent. While the relative difference in prevalence between the highest and the lowest wealth quintile has decreased among younger age groups (below 24 months), it has increased for children of preschool age (24 or above months) over time. For example, the relative gap (i.e., the ratio of the lowest to the highest wealth quintile) in prevalence among children aged below 12 months has decreased from 2.30 times in 2006 to 1.87 times in 2016. The ratio of stunting prevalence among the lowest to the highest wealth quintile has also decreased between 2006 and 2016 for children aged 12–23 months. For children between 24–35 months, the relative gap has slightly increased from 2.32 to 2.43. The gap was observed to be further higher for those from the 48–59 age-group, as prevalence among the lowest wealth quintile (54.5 percent) was 2.7 times that of the highest quintile (20.3 percent). It is also worth observing that the socioeconomic gradient in prevalence is higher for children from the older age groups.

**Figure A2:**
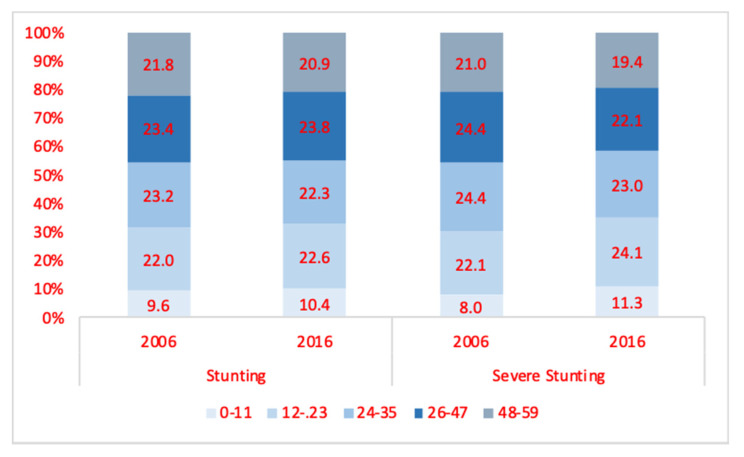
Distribution (%) of stunted and severely stunted children (below five years) by age-groups in India, NFHS, 2006, and 2016.

**Figure A3:**
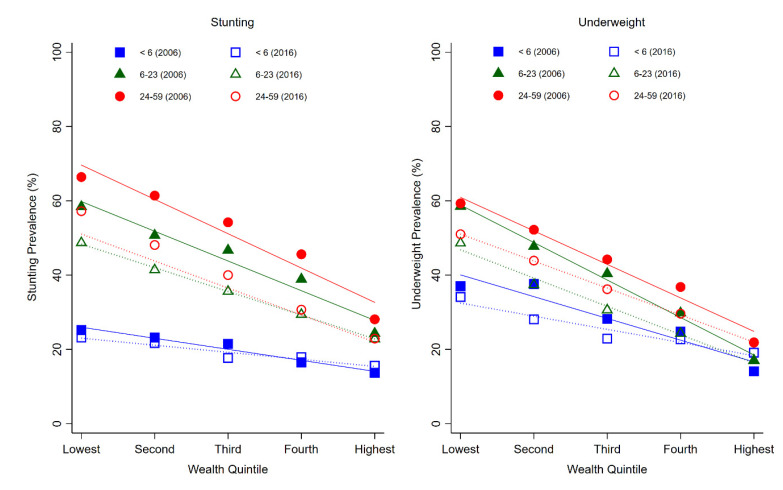
Prevalence of child anthropometric failures across age groups and wealth quintiles, India, NFHS 2006, and 2016.

In the case of severely stunted children, a similar age-specific socioeconomic pattern was observed but with intensified gaps between the lowest and the highest wealth quintile (Table A3 Appendix F). For example, in 2006, the prevalence among children aged 12–23 months from the lowest wealth quintile (39.1 percent) was 4.8 times those from the highest wealth quintile (8.2 percent). Across all age groups, the prevalence of severe stunting was much higher in the poorest households than in the richer households. The socioeconomic gradient in stunting prevalence was steeper in the case of children between 24–59 months compared to those between 0–23 months (Figure A2 Appendix D; Figure A3 Appendix E). 

**Figure 1:**
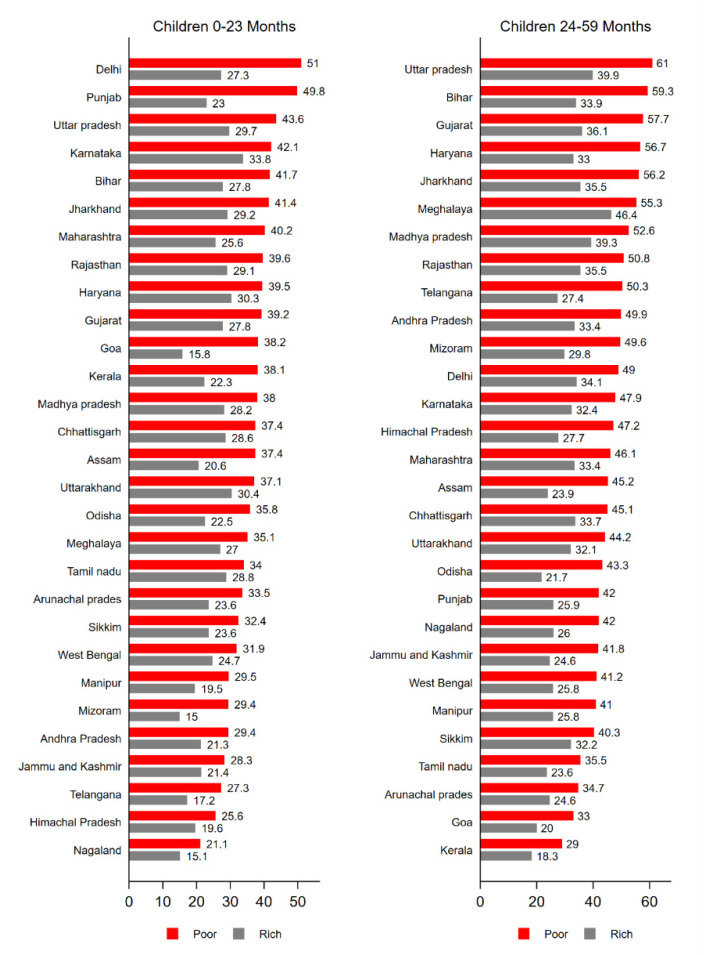
Prevalence of child stunting among children by age groups and wealth quintiles across the states, India, NFHS, 2016.

The observed differentials in stunting prevalence between children aged below and above two years was also evident across all states in India with higher prevalence among preschool children (24–59 months) from poor households (Figure 1). Across the states, the stunting prevalence among preschool children from the third or above wealth quintiles varied from 61.0 percent in Uttar Pradesh to 29.0 percent in Kerala in 2016. The prevalence among the same age-group children from poorer households (first or second wealth quintile) was highest for Delhi (51.0 percent) and lowest for Nagaland at 21.0 percent.

Table 3 presents estimates from multilevel logistic regressions for 2006 and 2016. In the fully adjusted model (Model 3), we found that compared to children below 12 months, the odds of stunting for children of older age groups were significantly higher. For 2016, model 3 shows that children between 36–47 months were about three times (OR: 2.91; 95% CI: 2.81; 3.00) more likely to be stunted as those below 12 months. The estimates from a fully adjusted model depict a decline in the likelihood of experiencing stunting for all age groups. For example, the OR for children in the 24–35 months age group was 4.01 (95% CI: 3.72; 4.30) in 2006 and 2.79 (295% CI: 2.69; 2.87) in 2016. Further, the OR for the 48–59 months age group also declined from 3.21 (95% CI: 2.99; 3.46) to 2.44 (95% CI: 2.36; 2.52). The econometric estimates for severe stunting also reflect reductions in relative risks of stunting for children from preschool age-groups (24 months or above) (Table A4 Appendix G).

Table 4 presents estimates from a multilevel logistic regression model with intersectional groups defined by child’s age and household’s wealth quintile. Across all intersectional groups, the relative risk of stunting was generally higher for children from the higher age groups and the lower wealth quintiles. Compared to children below 12 months from the wealthiest households, children from higher age-groups, and poorest households had a substantially higher likelihood of being stunted both in 2006 and 2016. For example, the value of OR for children between 24–35 months of age from poorest quintile was 8.37 (95% CI: 6.82; 10.27) in 2006 and 4.26 (95% CI: 3.88; 4.66) in 2016. The ORs were even higher for the poorest children in 36–47 months cohort both in 2006 (OR: 7.62; 95% CI: 6.23; 9.32) and 2016 (OR: 3.32; 95% CI: 3.09; 3.56). Among children aged 48–59 months from the poorest quintile, the odds of stunting were 6.00 (95% CI: 491; 7.34) and 3.63 (95% CI: 3.32; 3.98) in 2006 and 2016, respectively. However, it may be noted that the odds of stunting were also relatively higher for the poorest children from 12–23 months age group, i.e., 6.99 (95% CI: 5.70; 8.57) in 2006 and 3.92 (95% CI: 3.58; 4.29) in 2016. Across all intersectional groups, the over-time reduction in OR for stunting was relatively higher for children from the poorest quintile across all age groups. A similar econometric association was observed for severe stunting as well (Table A5 Appendix H)

## 4. Discussion

We present four salient findings that are highly relevant for policy discussions focused on improving child nutritional status in India and possibly other low-and middle-income countries (LMICs). First, the prevalence pattern of stunting across age groups shows a substantially higher burden among children aged 12 months and above. When classified into two age groups, a bulk of the total stunted children in India were in the age group 24–59 months. Strategies for achieving rapid improvements in nutritional status for children entering preschool age (24 or above months) and during the early adolescent phase are critical to avoid the risk of intergenerational transmission of undernutrition and poor health. Second, significant inequalities in the prevalence of stunting were observed among children from poorer households entering preschool age. Third, these inequalities have increased over time among children aged 24 months or above. However, no such rise in inequities was observed among younger children (below 24 months) across the wealth quintiles. Fourth, reductions (both absolute as well as relative) in child stunting over time were more prominent among children entering preschool age (24 months or above) compared to those below 24 months. In fact, improvements in child anthropometric failures were more apparent among children of preschool age-groups (24–35; 36–47; 48–59 months) from poorer households. This clearly implies that there is a huge potential for improving nutritional outcomes among preschool children.

Existing studies have asserted that immediate nutrition-specific interventions are important pathways to improve child nutritional status [21,22,23,24,25], and these interventions are more effective when introduced in the first 1000 days of life [26,27,28]. While the relevance of nutrition-specific interventions during the first two years of life cannot be contested, evidence on reversible nature of growth failures at later ages should also be emphasized. For example, a recent study analyzing longitudinal data from multiple LMICs found that children are both at risk of becoming stunted *and* potentially recovering post two years of age [9]. Randomized control trials (RCT) have claimed that improvements in the living environment and overall socioeconomic well-being can significantly improve anthropometric outcomes irrespective of children’s age [11,12]. These findings collectively call for revisiting the matrix for age-group based intervention strategies and devising effective policies to tackle the burden among the children entering preschool age (24 months or above). In fact, a systematic review also indicated the need for further research on understanding the standardization of the age group for pediatric interventions and RCTs [29].

Further, state-level estimates depict a substantially higher burden among poor children from states like Uttar Pradesh, Bihar, Haryana, Jharkhand, and Madhya Pradesh. The Government of India has launched several policies and programs to reduce the burden of child malnutrition, such as the *Balwadi* Nutrition Programme in 1975 and massive Integrated Child Development Services (ICDS). However, despite such long-standing programs, it is disconcerting to observe that every second child from the poorest households suffer from stunting. Even after experiencing a few episodes of very high economic growth between 2006 and 2016, the overtime improvements in child nutritional status have been sluggish. Besides socioeconomic and development disparities across states, communities, households, and individuals, such slow improvements are also attributable to poor multisectoral coordination at the implementation level. In this regard, recently launched POSHAN *Abhiyaan* (previously National Nutrition Mission) in 2018 aims to double the pace of reductions in child stunting, underweight and wasting in India. Our estimates suggest that it is critical to provide nutrition-sensitive and nutrition-related interventions to children of preschool age to attain desired reductions in POSHAN *Abhiyaan.*

Apart from direct nutrition interventions, improvements in socioeconomic and overall well-being have been identified as one of the most crucial drivers of child nutritional conditions occurring at various developmental stages [17]. Our estimates also suggest a huge gap in stunting prevalence between highest and lowest wealth quintiles among children from 24 months or above age groups. In fact, we found that this gap among children from higher age groups has increased over time. However, among children below 12 months and 12–24 months, wealth inequalities in stunting have decreased between 2006 and 2016. Large wealth variations in the prevalence of stunting among children of preschool age can perhaps be explained by increased direct exposure to household contextual living conditions as children grow older. The observed increase in wealth inequalities in stunting prevalence for children from higher age groups (i.e., above 23 months), but not among those below 24 months, indicates that research with robust study design to detect causality is needed to improve appropriate targeting for nutrition-sensitive interventions.

In addition, our findings from econometric analysis depict significant reductions in relative risk of failures among children of preschool age (24 months or above) from poorest households than those below 24 months. This implies that nutrition-specific and nutrition-sensitive interventions at a later age can potentially sustain the efforts made during the first 1000 days. Intuitively, the observed reductions in the odds of stunting among children from higher age-groups (24 months or above) can be partially attributed to the escalations in interventions at a younger age (i.e., first two years). However, in the Indian context, most of the essential interventions in the first 1000 days, such as antenatal care and immunization, are yet to elicit improvements both in terms of coverage as well as the quality of the service [30,31]. Further, the coverage of interventions under ICDS have also not shown any significant improvements among lower socioeconomic groups [32]. Besides, these government services are seldom utilized by richer households, thereby implying that a part of nutritional improvements is likely associated with household endowments (like wealth and better diet). In fact, recent studies have found relatively higher dietary diversity and a minimum acceptable diet among children from affluent households than those from the poorer economic backgrounds [33].

The present study has some limitations, as well. Firstly, the use of cross-sectional data restricts our estimations to the *prevalence* of anthropometric failures and does not allow us to make any claims about the *incidence* of these outcomes. The large proportion of stunted children between 24–59 months in our study represents a sum of continued growth failures that have occurred at an early age (i.e., the first 1000 days) and new incidences of failure at later childhood. While it is imperative to discern new incidence of child undernutrition across different age groups, the lack of longitudinal data on health and nutrition in India restricts such an examination. This is a key concern for researchers as well as policymakers. Studies based on longitudinal data suggest that children can become stunted or recover from stunting during birth to adolescence [9] and therefore, this limitation does not affect inferences made in this study. Third, the wealth index provided by NFHS was taken as a proxy for household income; therefore, any conclusion regarding poverty and deprivation can be sensitive to the adopted proxy for household economic status. Yet, evidence suggests that variables such as household wealth index are valid in general in population-based surveys [34]. Fourth, the analysis presented is not adjusted for seasonal variations; however, as a sensitivity analysis, we adjusted the model for calendar time, and it did not affect the arguments made in the paper. Finally, we had to exclude 23,181 children from the analysis due to missing age and anthropometric information.

## 5. Conclusions

Currently, the nutrition interventions under POSHAN Abhiyaan in India primarily target children below 24 months and a lesser focus is given on children of preschool age. While this policy advocacy to target malnutrition in the first two years of life is presumably due to steep declines in anthropometric z-scores prior to the age of 24 months [6], it is feasible that anthropometric failures continue, reverse, or newly occur throughout the entire childhood. While it is also worth noting that the observed reductions in stunting at higher age groups perhaps could be the outcome of accelerated nutrition interventions in the first 1000 days, greater analytical and policy attention for reducing burden among children aged 24–59 months is also critical to complement these strategic efforts targeting the first two years of life. It is imperative to strengthen the uptake and coverage of interventions under ICDS for children who belong to the preschool age bracket (24–59 months) both in rural and urban areas. Further, there is immense scope in terms of improving the quality of supplement diets for children above 24 months of age. Given the potential to catch-up growth at later age, a continuum in nutrition-related interventions till adolescence warrants policy attention.

## Figures and Tables

**Table 1:** Prevalence of child stunting by age groups, India, NFHS 2006, and 2016.

	2006		2016		Change	
Age Group (Months)	Prevalence (%)	95% CI	Prevalence (%)	95% CI	Absolute (% Points)	Relative ** (%)
0–11	24.9	[23.9; 25.8]	21.6	[21.2; 22.0]	3.3	13.2
12–23	52.4	[51.3; 53.4]	42.7	[42.2; 43.1]	9.7	18.5
24–35	55.9	[54.8; 56.9]	42.7	[42.2; 43.2]	13.2	23.6
36–47	54.4	[53.3; 55.4]	43.2	[42.8; 43.7]	11.2	20.6
48–59	50.6	[49.5; 51.7]	39.9	[39.5; 40.3]	10.7	21.1

**Note:** ** (Absolute Change/2006) × 100.

**Table 2:** Prevalence of child stunting by age groups and wealth quintiles, India, NFHS 2006, and 2016.

	2006		2016		Change	
Age Group (Months)	Prevalence (%)	95% CI	Prevalence (%)	95% CI	Absolute Change ** (% Points)	Relative Change *** (%)
0–11						
Lowest WQ	31.8	[29.2; 34.2]	27.8	[26.9; 28.6]	4	12.6
Second WQ	28.2	[25.8; 30.5]	23.6	[22.7; 24.4]	4.6	16.3
Third WQ	25.0	[22.8; 27.2]	20.0	[19.1; 20.8]	5	20.0
Fourth WQ	19.7	[17.8; 21.5]	17.9	[17.0; 18.8]	1.8	9.1
Highest WQ	13.8	[12.2; 15.6]	14.8	[13.9; 15.7]	−1	−7.2
Lowest/Highest *	2.30		1.87			
12–23						
Lowest WQ	65.7	[63.3; 68.2]	55.0	[54.1; 55.9]	10.7	16.3
Second WQ	57.7	[55.2; 60.1]	48.2	[47.2; 49.1]	9.5	16.5
Third WQ	53.9	[51.5; 56.3]	41.2	[40.2; 42.2]	12.7	23.6
Fourth WQ	46.2	[43.8; 48.4]	34.1	[33.1; 35.1]	12.1	26.2
Highest WQ	28.4	[26.3; 30.4]	26.6	[25.4; 27.6]	1.8	6.3
Lowest/Highest *	2.31		2.07			
24–35						
Lowest WQ	69.4	[66.9; 71.7]	58.2	[57.2; 59.0]	11.2	16.1
Second WQ	66.2	[63.8; 68.5]	49.0	[48.1; 49.9]	17.2	26.0
Third WQ	54.3	[52.0; 56.6]	41.1	[40.1; 42.1]	13.2	24.3
Fourth WQ	47.3	[45.1; 49.5]	31.9	[30.8; 32.9]	15.4	32.6
Highest WQ	30.1	[27.9; 32.2]	23.9	[22.8; 25.0]	6.2	20.6
Lowest/Highest *	2.32		2.43			
36–47						
Lowest WQ	67.6	[65.2; 69.9]	59.0	[58.1; 59.9]	8.6	12.7
Second WQ	59.4	[57.0; 61.9]	49.5	[48.6; 50.4]	9.9	16.7
Third WQ	55.9	[53.6; 58.1]	40.9	[39.9; 41.9]	15	26.8
Fourth WQ	47.4	[45.1; 49.6]	30.9	[29.9; 32.0]	16.5	34.8
Highest WQ	29.3	[27.2; 31.4]	24.4	[23.3; 25.4]	4.9	16.7
Lowest/Highest *	2.31		2.42			
48–59						
Lowest WQ	62.4	[59.9; 64.8]	54.5	[53.6; 55.4]	7.9	12.7
Second WQ	58.8	[56.4; 61.2]	45.9	[44.9; 46.8]	12.9	21.9
Third WQ	52.3	[49.9; 54.6]	37.9	[36.9; 38.9]	14.4	27.5
Fourth WQ	42.3	[40.0; 44.5]	29.5	[28.4; 30.5]	12.8	30.3
Highest WQ	24.9	[22.9; 26.8]	20.3	[19.3; 21.3]	7.9	12.7
Lowest/Highest *	2.51		2.68			

Note: * shows the relative difference between prevalence in lowest and highest wealth quintile, estimated as the ratio of lowest to highest wealth quintile: ** 2006–2016 *** (Absolute Change/2006) × 100.

**Table 3:** Econometric association between child stunting and age groups, India, NFHS 2006, and 2016.

Age Groups (Months)	2006		2016		Change
Model 1	OR	95% CI	OR	95% CI	(2006/2016)
0–11 ^®^	1.00		1.00		
12–23	3.16 ***	[2.94; 3.39]	2.61 ***	[2.52; 2.69]	1.21
24–35	3.61 ***	[3.36; 3.87]	2.62 ***	[2.53; 2.70]	1.38
36–47	3.43 ***	[3.20; 3.69]	2.76 ***	[2.67; 2.85]	1.24
48–59	2.97 ***	[2.76; 3.19]	2.38 ***	[2.30; 2.45]	1.25
**Model 2**					
0–11 ^®^	1.00		1.00		
12–23	3.39 ***	[3.15; 3.64]	2.72 ***	[2.64; 2.81]	1.25
24–35	3.84 ***	[3.57; 4.13]	2.73 ***	[2.63; 2.80]	1.41
36–47	3.86 ***	[3.39; 3.92]	2.86 ***	[2.77; 2.95]	1.35
48–59	3.15 ***	[2.93; 3.38]	2.43 ***	[2.35; 2.50]	1.30
**Model 3**					
0–11 ^®^	1.00		1.00		
12–23	3.51 ***	[3.26; 3.77]	2.77 ***	[2.68; 2.85]	1.27
24–35	4.01 ***	[3.72; 4.30]	2.79 ***	[2.69; 2.87]	1.44
36–47	3.76 ***	[3.50; 4.04]	2.91 ***	[2.81; 3.00]	1.29
48–59	3.21 ***	[2.99; 3.46]	2.44 ***	[2.36; 2.52]	1.32

Estimates are *** significant at 0.01 level. Odds Ratios are estimated from Multilevel Regression (three-level) adjusting for State, and Village Level Random effects. ^®^ refers to the reference category. Model 1 adjusts for age and sex. Model 2 in addition to age and sex, adjusts for socioeconomic variables including wealth quintile, social group, religion, place of residence. Model 3 further adjusts for maternal covariates like Mother’s Body Mass Index BMI, Mother’s height and Mother’s education.

**Table 4:** Multilevel logistic regression-based odds ratio of child stunting across age groups (in months) and wealth quintiles, NFHS 2006 and 2016.

	2006		2016		Change
Age * Wealth Quintile	OR	95% CI	OR	95% CI	(2006/2016)
(0–11) * WQ5 ^®^	1.00		1.00		
(0–11) * WQ4	1.22	[1.00;1.49]	1.02	[0.92;1.12]	1.20
(0–11) * WQ3	1.43 ***	[1.16;1.74]	1.03	[0.93;1.13]	1.39
(0–11) * WQ2	1.48 ***	[1.20;1.82]	1.13 **	[1.03;1.25]	1.31
(0–11) * WQ1	1.69 ***	[1.37;2.08]	1.28 ***	[1.16;1.40]	1.32
(12-23) * WQ5	2.65 ***	[2.20;3.20]	1.97 ***	[1.79;2.17]	1.35
(12-23) * WQ4	3.80 ***	[3.16;4.57]	2.38 ***	[2.17;2.61]	1.60
(12-23) * WQ3	4.93 ***	[4.08;5.97]	2.88 ***	[2.63;3.15]	1.71
(12-23) * WQ2	5.72 ***	[4.70;6.96]	3.36 ***	[3.07;3.68]	1.70
(12-23) * WQ1	6.99 ***	[5.70;8.57]	3.92 ***	[3.58;4.29]	1.78
(24–35 ) * WQ5	2.75 ***	[2.28;3.32]	1.75 ***	[1.59;1.93]	1.57
(24–35 ) * WQ4	4.28 ***	[3.57;5.14]	2.22 ***	[2.02;2.43]	1.93
(24–35 ) * WQ3	5.55 ***	[4.60;6.70]	2.83 ***	[2.59;3.10]	1.96
(24–35 ) * WQ2	7.14***	[5.86;8.70]	3.50 ***	[3.20;3.83]	2.04
(24–35 ) * WQ1	8.37 ***	[6.82;10.2]	4.26 ***	[3.88;4.66]	1.96
(36–47 ) * WQ5	2.63 ***	[2.17;3.17]	1.81 ***	[1.65;2.00]	1.45
(36–47 ) * WQ4	4.27 ***	[3.56;5.14]	2.30 ***	[2.10;2.52]	1.86
(36–47 ) * WQ3	5.31 ***	[4.40;6.40]	2.96 ***	[2.70;3.24]	1.79
(36–47 ) * WQ2	6.13 ***	[5.04;7.46]	3.71 ***	[3.39;4.06]	1.65
(36–47 ) * WQ1	7.62 ***	[6.23;9.32]	4.41 ***	[4.03;4.83]	1.73
(48–59 ) * WQ5	1.93 ***	[1.59;2.34]	1.50 ***	[1.36;1.66]	1.29
(48–59 ) * WQ4	3.74 ***	[3.11;4.50]	1.93 ***	[1.76;2.12]	1.94
(48–59 ) * WQ3	4.93 ***	[4.08;5.95]	2.56 ***	[2.33;2.80]	1.93
(48–59 ) * WQ2	5.68 ***	[4.67;6.91]	3.10 ***	[2.84;3.40]	1.83
(48–59 ) * WQ1	6.00 ***	[4.91;7.34]	3.63 ***	[3.32;3.98]	1.65

Note: Estimates are * significant at 0.10 ** at 0.05 level *** at 0.01 level. Odds Ratios are estimated from Multilevel Regression (three-level) adjusting for State, and Village Level Random effects. ^®^ refers to the reference category. The regression Model is adjusted for age, sex, socioeconomic variables, including wealth quintile, social group, religion, place of residence, and maternal covariates like mother’s BMI, mother’s height, and mother’s education. WQ-Wealth Quintile.

## Appendix B

**Table A1:** Prevalence of severe stunting among children (below five years) by age groups, India, NFHS 2006 and 2016.

	2006		2016		Change	
Age Group (Months)	Prevalence (%)	95% CI	Prevalence (%)	95% CI	Absolute (% Points)	Relative ** (%)
0–11	10.3	[09.6; 10.9]	09.9	[09.7; 10.2]	0.4	0.04
12–23	26.0	[25.1; 26.9]	19.3	[18.9; 19.6]	6.7	02.6
24–35	29.0	[28.0; 29.9]	18.6	[18.3; 18.9]	10.4	35.9
36–47	27.9	[27.0; 28.9]	17.0	[16.7; 17.4]	10.9	39.1
48–59	24.1	[23.2; 25.0]	15.7	[15.3; 16.]	8.4	34.9

**Note:** ** (Absolute Change/2006) × 100.

## Appendix C

**Table A2:** Prevalence of anthropometric failures among children by Age groups, India, NFHS 2006 and 2016.

Age Group	Stunting (%)			
	2006	2016	Absolute Change * (% Points)	Relative Change ** (%)
0–23 months	39.2	32.6	6.6	16.8
	[38.4; 39.9]	[32.3; 32.9]		
24–59 months	53.6	42.0	11.6	21.6
	[52.9; 54.2]	[41.7; 42.2]		

**Note:** * 2006–2016 ** (Absolute Change/2006) × 100.

## Appendix F

**Table A3:** Prevalence of severe stunting among children (below five years) by age groups and wealth quintiles, India, NFHS, 2016.

	2006		2016		Change	
Age Group (Months)	Prevalence (%)	95% CI	Prevalence (%)	95% CI	Absolute Change * (% Points)	Relative Change** (%)
0–11						
Lowest WQ	13.3	[11.4; 15.1	13.2	[12.5; 13.8]	0.1	0.8
Second WQ	12.1	[10.3; 13.7]	10.3	[09.6; 10.8]	1.8	14.9
Third WQ	10.3	[08.7; 11.8]	08.8	[08.2; 09.4]	1.5	14.6
Fourth WQ	08.2	[06.8; 09.5]	08.6	[07.9; 09.3]	−0.4	−4.9
Highest WQ	04.7	[03.7; 05.7]	06.9	[06.3; 07.6]	−2.2	−46.8
Lowest/Highest ^©^	2.83		1.91			
12–23						
Lowest WQ	39.1	[36.6; 41.6]	28.8	[27.9; 29.6]	10.3	26.3
Second WQ	28.9	[26.6; 31.2]	21.6	[20.8; 22.4]	7.3	25.3
Third WQ	26.2	[24.1; 28.3]	16.7	[15.9; 17.4]	9.5	36.3
Fourth WQ	19.1	[17.3; 20.9]	13.6	[12.8; 14.3]	5.5	28.8
Highest WQ	08.2	[06.9; 09.4]	10.6	[09.8; 11.4]	−2.4	−29.3
Lowest/Highest ^©^	4.76		2.72			
24–35						
Lowest WQ	42.9	[40.3; 45.4]	30.7	[29.8; 31.5]	12.2	28.4
Second WQ	36.4	[33.9; 38.8]	21.9	[21.1; 22.7]	14.5	39.8
Third WQ	26.5	[24.5; 28.5]	14.9	[14.2; 15.6]	11.6	43.8
Fourth WQ	19.8	[18.0; 21.6]	11.5	[10.8; 12.2]	8.3	41.9
Highest WQ	09.1	[07.7; 10.4]	08.1	[07.3; 08.7]	1	11.0
Lowest/Highest ^©^	4.71		3.79			
36–47						
Lowest WQ	39.7	[37.3; 42.1]	28.7	[27.8; 29.4]	11	27.7
Second WQ	31.7	[29.4; 34.0]	19.5	[18.7; 20.2]	12.2	38.5
Third WQ	28.9	[26.8; 30.9]	13.7	[13.0; 14.4]	15.2	52.6
Fourth WQ	18.8	[17.0; 20.5]	09.5	[08.9; 10.2]	9.3	49.5
Highest WQ	11.3	[09.8; 12.7]	06.5	[05.9; 07.1]	4.8	42.5
Lowest/Highest ^©^	3.51		4.41			
48–59						
Lowest WQ	34.9	[32.5; 37.4]	25.6	[24.8; 26.3	9.3	26.6
Second WQ	30.3	[28.0; 32.6]	17.7	[16.9; 18.4]	12.6	41.6
Third WQ	22.5	[20.6; 24.5]	12.9	[12.2; 13.6]	9.6	42.7
Fourth WQ	16.2	[14.5; 17.9]	09.0	[08.4; 09.6]	7.2	44.4
Highest WQ	07.4	[06.1; 08.5]	06.6	[05.9; 07.2]	0.8	10.8
Lowest/Highest ^©^	4.71		3.88			

Note: ^©^ shows the relative difference between prevalence in lowest and highest wealth quintile, estimated as the ratio of lowest to highest wealth quintile: * Absolute Difference between 2006 and2016 ** (Absolute Change between 2006–2016) /2006) × 100 × 100.

## Appendix G

**Table A4:** Econometric association between severe stunting among children (below five years) and age groups, India, NFHS 2006, and 2016.

Age Groups (Months)	2006		2016		Change
Model 1	OR	95% CI	OR	95% CI	(2006/2016)
0–11 ^®^	1.00		1.00		
12–23	2.80 ***	[2.54; 3.09]	2.19 ***	[2.09; 2.28]	1.28
24–35	3.22 ***	[2.93; 3.55]	2.06 ***	[1.97; 2.16]	1.56
36–47	3.00 ***	[2.73; 3.31]	1.88 ***	[1.80; 1.97]	1.60
48–59	2.43 ***	[2.20; 2.68]	1.71 ***	[1.62; 1.78]	1.42
**Model 2**					
0–11 ^®^	1.00		1.00		
12–23	2.92 ***	[2.65; 3.21]	2.23 ***	[2.13; 2.33]	1.31
24–35	3.34 ***	[3.04; 3.67]	2.09 ***	[2.00; 2.18]	1.60
36–47	3.09 ***	[2.81; 3.40]	1.93 ***	[1.80; 1.96]	1.60
48–59	2.49 ***	[2.26; 2.75]	1.69 ***	[1.61; 1.76]	1.47
**Model 3**					
0–11 ^®^	1.00		1.00		
12–23	2.99 ***	[2.71; 3.29]	2.24 ***	[2.14; 2.33]	1.33
24–35	3.42 ***	[3.11; 3.76]	2.09 ***	[2.01; 2.19]	1.64
36–47	3.14 ***	[2.86; 3.46]	1.87 ***	[1.78; 1.95]	1.68
48–59	2.49 ***	[2.27; 2.76]	1.65 ***	[1.58; 1.73]	1.51

Note: Estimates are *** significant at 0.01 level. Odds Ratios are estimated from Multilevel Regression (three-level) adjusting for State, and Village Level Random effects. ^®^ refers to reference category. Model 1 adjusts for age and sex. Model 2, in addition to age and sex, adjusts for socioeconomic variables including wealth quintile, social group, religion, place of residence. Model 3 further adjusts for maternal covariates like Mother’s BMI, Mother’s height, and Mother’s education.

## Appendix H

**Table A5:** Multilevel logistic regression-based odds ratio of severe stunting among children (below five years) across age groups (in months) and wealth quintiles, NFHS 2006, and 2016.

	2006		2016		Change
Groups	OR	95% CI	OR	95% CI	(2006/2016)
(0–11) * WQ5 ^®^	1.00		1.00		
(0–11) * WQ4	1.27	[0.94; 1.73]	1.02	[0.88; 1.17]	1.25
(0–11) * WQ3	1.55 ***	[1.14; 2.10]	0.99	[0.86; 1.14]	1.57
(0–11) * WQ2	1.60 ***	[1.18; 2.17]	1.07	[0.93; 1.22]	1.50
(0–11) * WQ1	1.77 ***	[1.30; 2.40]	1.21 ***	[1.06; 1.38]	1.46
(12–23) * WQ5	2.08 ***	[1.55; 2.79]	1.58 ***	[1.38; 1.82]	1.32
(12–23) * WQ4	3.09 ***	[2.34; 4.08]	1.76 ***	[1.54; 2.01]	1.76
(12–23) * WQ3	4.45 ***	[3.37; 5.88]	2.04 ***	[1.80; 2.32]	2.18
(12–23) * WQ2	4.91 ***	[3.70; 6.50]	2.54 ***	[2.23; 2.88]	1.93
(12–23) * WQ1	6.89 ***	[5.19; 9.16]	3.18 ***	[2.80; 3.61]	2.17
(24–35 ) * WQ5	2.11 ***	[1.57; 2.84]	1.07	[0.92;1.25]	1.97
(24–35 ) * WQ4	3.38 ***	[2.57; 4.46]	1.51 ***	[1.32; 1.73]	2.24
(24–35 ) * WQ3	4.78 ***	[3.63; 6.30]	1.84 ***	[1.61; 2.09]	2.60
(24–35 ) * WQ2	6.64 ***	[5.02; 8.79]	2.52 ***	[2.22; 2.86]	2.63
(24–35 ) * WQ1	7.88 ***	[5.94; 10.46]	3.23 ***	[2.84; 3.66]	2.44
(36–47 ) * WQ5	2.13 ***	[1.59; 2.87]	1.04	[0.90; 1.21]	2.05
(36–47 ) * WQ4	3.32 ***	[2.52; 4.37]	1.27 ***	[1.11; 1.46]	2.61
(36–47 ) * WQ3	4.43 ***	[3.36; 5.84]	1.63 ***	[1.43; 1.86]	2.72
(36–47 ) * WQ2	5.50 ***	[4.15; 7.28]	2.19 ***	[1.93; 2.49]	2.51
(36–47 ) * WQ1	7.16 ***	[5.40; 9.50]	2.91 ***	[2.56; 3.30]	2.46
(48–59 ) * WQ5	1.40 ***	[1.02; 1.91]	1.04	[0.90; 1.21]	1.35
(48–59 ) * WQ4	2.61 ***	[1.97; 3.45]	1.15	[1.00; 1.32]	2.27
(48–59 ) * WQ3	3.48 ***	[2.63; 4.60]	1.55 **	[1.36; 1.76]	2.25
(48–59 ) * WQ2	4.79 ***	[3.62; 6.35]	1.91 ***	[1.68; 2.17]	2.51
(48–59 ) * WQ1	5.74 ***	[4.32; 7.63]	2.47 ***	[2.17; 2.80]	2.32

Note: Estimates are * significant at 0.10 ** at 0.05 level *** at 0.01 level. Odds Ratios are estimated from Multilevel Regression (three-level) adjusting for State, and Village Level Random effects. ^®^ refers to the reference category. The regression Model is adjusted for age, sex, socioeconomic variables including wealth quintile, social group, religion, place of residence, and maternal covariates like mother’s BMI, mother’s height, and mother’s education.
